# Case Report: Personalized peptide-based immunization in an advanced-stage prostate cancer patient with bone metastasis

**DOI:** 10.3389/fonc.2025.1596315

**Published:** 2025-07-22

**Authors:** Nils Heinrich Thoennissen, Mari Carmen Martos-Contreras, Mehdi Manoochehri, Mauro Nogueira, Franziska Bremm, Jan Dörrie, Jan Christoph, Meik Kunz, Wolfgang Schönharting

**Affiliations:** ^1^ University of Munich, Medical Clinic III, Munich, Germany; ^2^ Immuneo Therapeutics GmbH, University Clinic Brandenburg, Brandenburg an der Havel, Germany; ^3^ Department of Bioinformatics, Biocenter, University of Würzburg, Würzburg, Germany; ^4^ Department of Dermatology, Friedrich-Alexander-Universität Erlangen-Nürnberg, Universitätsklinikum Erlangen, Erlangen, Germany; ^5^ Comprehensive Cancer Center Erlangen European Metropolitan Area of Nürnberg (CCC ER-EMN), Erlangen, Germany; ^6^ Deutsches Zentrum Immuntherapie (DZI), Erlangen, Germany; ^7^ Bavarian Cancer Research Center (BZKF), Erlangen, Germany; ^8^ Chair of Medical Informatics, Friedrich-Alexander University (FAU) of Erlangen-Nürnberg, Erlangen, Germany; ^9^ AG Bio-Medical Data Science, Martin-Luther-Universität Halle-Wittenberg, Halle, Germany

**Keywords:** immunotherapy, neoantigen, peptide, prostate cancer, case report

## Abstract

Neoantigens, which are recognized as non-self and trigger an immune response, are novel antigens generated by tumor cells. Here, we report a *de novo* metastatic hormone-sensitive prostate cancer (mHSPC) case, which benefited from our personalized peptide immunization named BioInformatic Tumor Address Peptides (BITAP) in a monotherapeutic setting. Our in-house bioinformatics pipeline involved identifying somatic variations, analyzing their expression, and computationally predicting novel epitopes from both metastatic and primary tumors, separately. As stand-alone therapy, the patient has been administered multiple injections of two peptide pools (BITAP-1 and BITAP-2). Several months following immunizations, a significant regression of both metastatic and primary tumor lesions was recorded along with low-level of prostate-specific antigen (PSA). Besides mild and short-lasting local and systemic reactions, no serious treatment-related adverse effects were reported by the patient. In conclusion, this case suggests that BITAP immunization is feasible and safe, and may present an immunotherapeutic approach inducing sustainable tumor regressions in mHSPC patients.

## 1 Introduction

In Western countries, prostate cancer (PrCa) is the most commonly diagnosed cancer and one of the leading causes of cancer-related death among men (1). Over the past years, some progress has been made for the treatment of metastatic PrCa (2), and the 5-year survival for distant stage PrCa improved from 28.7% to 32.3% (3). Although targeting bone metastases in prostate cancer leads to improving clinical outcome (4), currently, there are no effective treatments for these patients (5).

During the last decade, immunotherapy has shown clinical benefit across various tumor entities such as melanoma, lung cancer, and renal cell carcinoma (6). Cancer vaccines have been under development over the past century, and shown promise by priming T cell and prompting antigen-specific responses targeting tumor antigens, including tumor-associated antigens (TAAs) and tumor-specific antigens (TSAs). Personalized therapeutic cancer vaccines have recently attracted a lot of attention due to the developments in next generation sequencing technologies and a deeper understanding of neoantigens production (7).

Unfortunately, bone metastatic PrCa is considered an ‘immune-cold’ tumor, resulting in poor immune checkpoint therapy (ICT) responses (8). Despite advances in the management of mHSPC, the emergence of castration resistance is unavoidable, and many patients develop metastatic castration-resistant disease (mCRPC). Numerous clinical trials have been completed or are ongoing to assess various immunotherapeutic approaches for patients with metastatic PrCa (9). This clinical case report sheds light on the application of a personalized peptide-based immunization approach, referred to as BITAP, in a PrCa patient with bone metastasis.

## 2 Case description

A 72-year-old Caucasian male was admitted to the urologist on 04^th^ October 2022, complaining of urinary urgency, interrupted urination, nocturia, and erectile dysfunction. At this time, prostate-specific antigen (PSA) level was measured to be 99.8 ng/mL. The Magnetic Resonance Imaging (MRI) examination showed a moderately enlarged prostate, with an extensive, organ-crossing and infiltrating mass on the left side of prostate organ, highly suspicious for PrCa. The prostate-specific membrane antigen positron emission tomography (PSMA-PET) examination revealed significant involvement of the middle of the prostate gland and the apex on the left periphery, with infiltration extending to the left seminal vesicles. No evidence of PSMA-positive lymph node metastases was documented. However, a total of 32 multilocular sclerotic bone metastases were reported. To finally confirm the suspected diagnosis of advanced PrCa, biopsies were taken from bone metastases in the area of the lateral mass of the left sacrum. Pathological assessment of the biopsies from metastatic tumor revealed a strong expression of cytokeratin, NKX3.1 and P504S which prove malignant prostatic origin of the metastatic tumors. Secondary biopsies were also taken from both the primary tumors (according to the pathologists letter; no biopsy data shown) and non-tumor regions of the prostate. The pathological assessment revealed a Gleason pattern of 4 + 4, corresponding to a Gleason score of 8, indicating high-grade prostatic adenocarcinoma.

## 3 Therapeutic intervention

**Figure 1:**
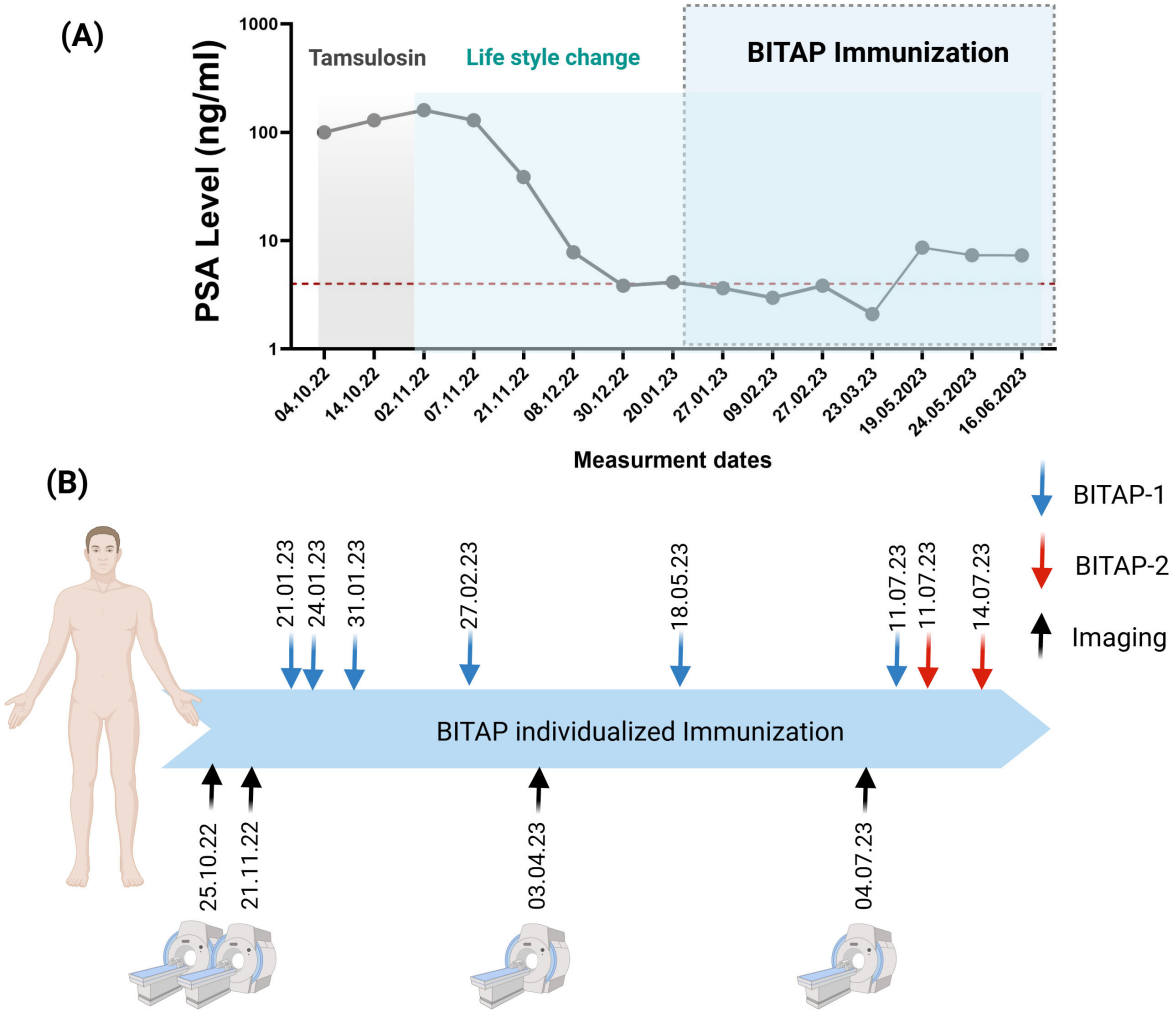
**(A)** The PSA levels of the patient during the time of patient diagnosis and treatment. **(B)** Timeline for the immunization process of a prostate cancer patient using multi-peptide BITAP pools. The patient has been receiving different BITAP peptide pools (BITAP-1 and BITAP-2) over several months. Each BITAP pool was developed using a new NGS (next-generation sequencing) analysis: one derived from metastatic tumor (BITAP-1) and the other from primary tumor (BITAP-2). Created with BioRender.com.

The patient was already symptomatically treated for 4 weeks of Tamsulosin to manage lower urinary tract symptoms associated with prostate enlargement (see **Figure 1A**). Immediately, following the histological diagnosis of *de novo* mHSPCA, evidence-based first-line systemic therapy with androgen deprivation therapy (ADT) plus abiraterone was strongly recommended to the patient. However, patient explicitly refused this regimen due to his concerns about potential side effects. Instead, the patient opted for lifestyle changes, including diet with addition of fermented soy products, and strictly avoiding smoking and alcohol consumption. Surprisingly, this individual lifestyle approach led to a continuous drop in PSA levels over the following 10 weeks (see **Figure 1A**). However, follow-up whole-body MRI after the initial diagnosis revealed progressive bone metastases suggesting regulatory effects of the soy products only on PSA kinetics without any anti-tumor activity. It has already been reported that soy products may alter PSA levels in untreated PrCa patients (10, 11). Still explicitly refusing evidence-based treatments, patient reached out then for personalized immunization by BITAP. After being given detailed information about the experimental character of this novel treatment approach, the patient received the first prime dose of BITAP-1 by subcutaneous injection (see **Figure 1A**). The next prime doses of BITAP-1 were applied 3, 10 and 36 days after the first injection. The patient received two boost injections 3 and 5 months after priming. The immunization process of the prostate cancer patient using multi-peptide BITAP pools in a monotherapeuitc setting is shown in **Figure 1B**.

## 4 Methods

### 4.1 Next generation sequencing and preparation of immunization pool

Genomic DNA and RNA extracted from metastatic and primary tumor tissues, along with genomic DNA obtained from blood samples, were subjected to next-generation sequencing (NGS) analysis (**Supplementary Figure 1**). The selection of neoantigen-containing peptides in the immunization peptide pool was performed according to our in-house BITAP analysis pipeline and using sequencing data of tumor, normal and blood samples. Neoantigen-containing peptides refer to synthetic long peptides that include 8–15-mer neoantigenic sequences derived from tumor-specific mutations. These are extended at the N- or C-terminal to enhance immunogenicity and presentation by both MHC class I and II molecules. The peptides were then produced through chemical synthesis at >90% purity to generate the immunization peptide pool (Intavis Peptide Services GmbH, Tübingen, Germany). The details of the immunization peptide pool preparation are explained in the **Supplementary Materials**.

### 4.2 T-cell responses of BITAP

**Figure 2:**
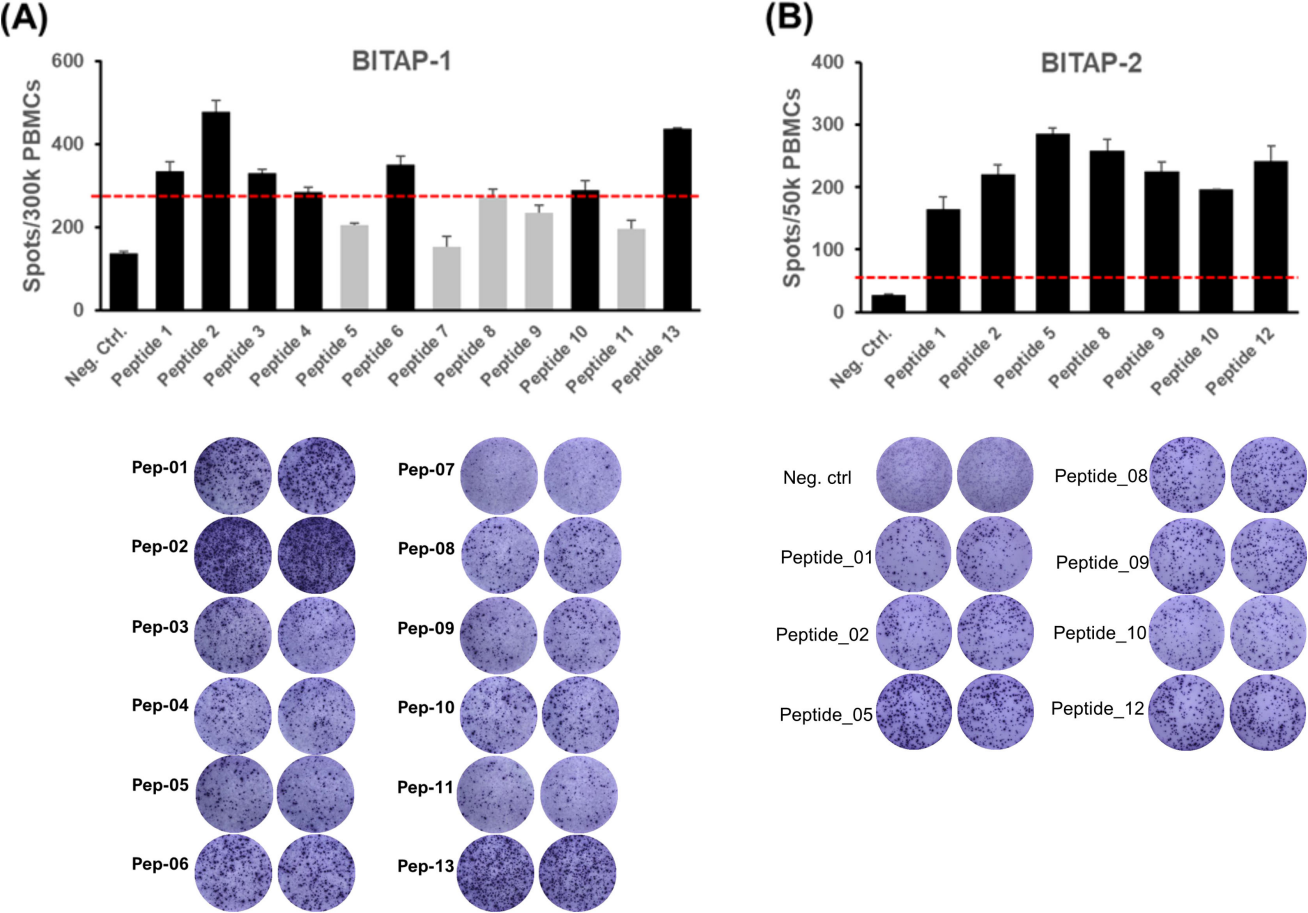
Immunogenicity testing of BITAP peptides using ELISpot assay. Blood was taken from the patient prior to the first application of the respective peptide pool, and a 10–12 days ex vivo pre-stimulation was performed. Afterward, the numbers of specific cells against the individual peptides were determined. The red dotted lines indicate the 2-fold negative control values. Seven out of 12 peptides from BITAP-1 were immunogenic **(A)**. Testing of BITAP-2 peptides using ELISpot assay revealed immunogenicity of all 7 **(B)**. The red line represents the threshold line for results to be considered positive. The ELISpot response was considered positive if it showed at least a two-fold increase over the negative control and exceeded 50 spot-forming units (SFUs) per 1×10^6^ PBMCs. **(A)** shows mean spot counts from duplicate wells per peptide condition. **(A, B)** display representative images of ELISpot wells corresponding to the same experiment. PBMCs were collected before the first BITAP injection for each cocktail.

The immunogenicity of each peptide was analyzed in the patient’s peripheral blood mononuclear cells (PBMCs) using an IFN-γ ELISpot assay (11). The ELISpot results were considered positive when the numbers of spots were at least 2-fold above the negative control (medium) and with a minimum of 50 detected spots. Accordingly, strong responses were shown in 7 out of 12 (~ 58% positivity) peptides from BITAP-1 which were prepared by the analysis of bone metastases (**Figure 2A**). The BITAP-2 peptide pool was prepared by the evaluation of biopsies from primary tumor and non-tumor tissues of prostate. Initially, the 12 predicted epitopes were combined into 7 peptides and tested using ELISpot assays (**Figure 2B**). Subsequently, these peptides underwent Good Manufacturing Practice-like (GMP-like) production and were incorporated into the BITAP-2 pool for injection into the patient. The dosing regimens for BITAP-1 and BITAP-2 were aligned with those used in our previous case report as well as prior clinical trials investigating personalized neoantigen peptide vaccines (12).

## 5 Clinical outcome

**Figure 3:**
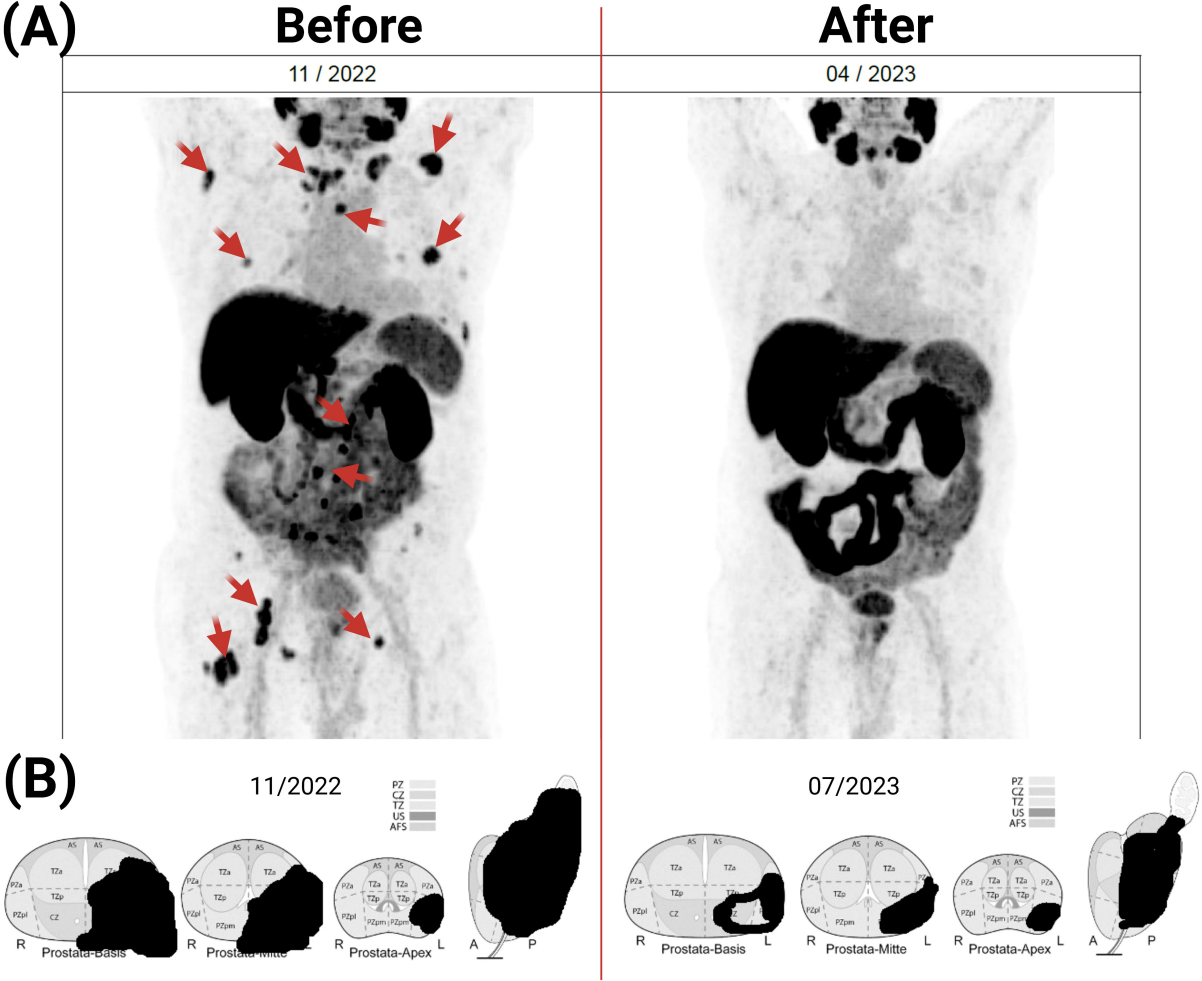
Effect of BITAP immunization on the metastatic and primary tumor lesions. The patient examinations before and after BITAP immunization treatment was done by PSMA-PET CT **(A)** and MRI **(B)**. The MRI of the prostate revealed an additional 40% reduction in primary tumor volume at five months after BITAP immunization.

Approximately two months after the first dose of BITAP-1, patient examination using PSMA-PET showed a clear regression of multiple bone metastases compared to the previous examination, which had been performed before starting the BITAP immunization (**Figure 3A**). The PSMA-expressing metastases still showed a low PSMA expression, which indicates a minimal residual viability. Additionally, no evidence of new bone metastases was apparent. Another imaging analysis of the primary tumor using MRI at five months following the start of BITAP immunization showed an additional regression of 40% in the primary tumor mass (**Figure 3B**). After the patient had received multiple primary and booster injections of BITAP-1, the BITAP-2 immunization was started (**Figure 1B**). The patient received routine clinical follow-up during and after the immunization cycles. Monitoring included vital signs, and PSA levels at regular intervals. Due to ethical constraints, no experimental testing beyond standard-of-care diagnostics was performed.

## 6 Treatment-related adverse effects

Beside weekly doctoral follow-ups, the patient was encouraged to actively participate in his treatment journey by promptly documenting and reporting any new or unusual symptoms. The treatment-related adverse effect reported and documented by the patient included only short-term local reactions after BITAP administration such as burning sensation and redness at the subcutaneous injection site. In addition, the patient also experienced mild systemic symptoms, including increased night sweats, during the first two days following each BITAP injection.

## 7 Discussion

The presented case study highlights a promising avenue in metastatic prostate cancer therapy through the application of a personalized neoantigen cancer immunization. The patient’s response to the immunization peptide pool (BITAP), as evidenced by the considerable regression of metastatic and primary tumor lesions along with maintenance of low-level PSA, suggests the potential efficacy of this immunotherapeutic approach. The patient implemented individual lifestyle changes during the course of treatment, which may have influenced PSA kinetics; therefore, the observed PSA reduction cannot be attributed solely to BITAP immunotherapy, and future controlled studies will be necessary to define these effects.

The positive outcome of this case study aligns with the growing evidence on neoantigens as key targets for cancer vaccines (13, 14). Emerging from tumor-specific alterations, neoantigens could trigger a highly specific immune response against the cancer cells while minimizing damage to the healthy tissues (15). As shown, the patient’s positive response to the BITAP immunization supports the concept that neoantigens can indeed serve as potent antitumor stimulator for immune recognition and activation, while triggering only minimal and short-lasting immune-related side effects. However, it is important to highlight the complexity of the immune system and the potential variability in individual responses to neoantigen-based therapies. Various factors such as tumor heterogeneity, immune status, and the tumor microenvironment can influence treatment outcomes (16). The potential for synergy between BITAP peptide vaccination and immune checkpoint inhibitors as combining these modalities might enhance antitumor immunity by both broadening T cell responses and overcoming immune evasion. Despite the positive outcome in this case, the broader applicability and consistent response rates across a diverse patient population need to be further investigated.

The duration of the observed response and the potential for long-term remission also warrant consideration. Monitoring the patient’s progress over a longer period and exploring mechanisms that contribute to the observed immune responses will provide valuable insights into the durability of BITAP immunization efficacy. The durability of immune responses may be limited by tumor escape mechanisms such as neoantigen loss or immune suppression. While no post-treatment biopsy was available to assess these changes in this case, future studies should include longitudinal tissue analyses to better understand resistance and guide re-immunization strategies. Additionally, this case study suggests that a combination of BITAP therapy with other treatment modalities, such as immune checkpoint inhibitors could potentially enhance the therapeutic effect by leveraging multiple aspects of the immune response and tumor biology. As this case study contributes to the evolving landscape of prostate cancer immunotherapy, it prompts the need for larger clinical trials to validate its findings and establish its place within the current treatment paradigm.

## 8 Conclusions

This case illustrates the potential of personalized peptide-based immunization in managing advanced PrCa patients with bone metastasis. The BITAP therapy yielded a positive response in this patient, suggesting it could be an innovative and promising approach for other patients with similar conditions. However, more clinical data is needed to conclusively validate these findings. In addition, identifying patients who would benefit most from BITAP therapy through companion diagnostic markers is essential for clinical translation. Therefore, future studies should focus on defining predictive biomarkers, such as neoantigen load, T cell repertoire, and tumor immune microenvironment to optimize patient selection. The potential benefits and side effects of this individualized immunotherapy approach also need further investigation. Importantly, ethical and regulatory standards must be stringently adhered to in the deployment of such personalized treatments.

## 9 Patient perspective

From the patient’s perspective, the decision to undergo the BITAP personalized neoantigen cancer immunization was a significant step in managing his disease. The observed regression of tumor lesions and stabilization of PSA levels were interpreted by the patient as encouraging indicators of progress. These outcomes contributed to a sense of relief and optimism about the potential of this novel intervention. The patient emphasized the importance of open communication and collaborative decision-making with healthcare providers, which he felt was integral to his care experience.

## Data Availability

The datasets presented in this article are not readily available because due to data protection regulations, we are not allowed to share the sequencing data generated in this study. Access to the data is restricted to authorized personnel involved in the research, and all patient information is anonymized to ensure privacy and confidentiality. Requests to access the datasets should be directed to Meik Kunz (meik.kunz@fau.de) and Mari Carmen Martos-Contreras (mari.martos@immuneo-therapeutics.de).
