# Supplementary material for: Case Report: Personalized peptide-based immunization in an advanced-stage prostate cancer patient with bone metastasis
Source: Front Oncol. 2025 Jul 22;15:1596315. doi: 10.3389/fonc.2025.1596315 (PMC12321524; doi:10.3389/fonc.2025.1596315)
Supplement: Supplementary file 1 [file DataSheet1.docx]

Supplementary Material

# Supplementary Data

**Material and methods**

**Next Generation Sequencing and Bioinformatics**

Tumor tissues from bone metastases, primary tumor and non-tumor inflammatory regions of the prostate was snap-frozen in liquid nitrogen immediately after surgical resection and stored at –80°C until nucleic acid extraction for next-generation sequencing (NGS). The first BITAP was generated from the metastatic tumor tissue whereas primary tumor and non-tumor inflammatory regions of the prostate were used for the second BITAP. For both, DNA and RNA were isolated from the biopsy tissue samples (AllPrep DNA/RNA Mini Kit; Qiagen) which were already evaluated by pathologist. DNA from EDTA blood sample was isolated (QIA Symphony DSP DNA Mini Kit 96; Qiagen, Hilden, Germany) and considered as normal tissue in following analysis (**Suppl.** **Figure 1**). Library preparation for Whole-Exome-Sequencing (WES) was performed using Twist Human Core Exome kit with RefSeq and Mitochondrial Panel (Twist Bioscience, San Francisco, USA). Library preparation for RNA-seq (RNA sequencing) was carried out by KAPA RNA HyperPrep Kit with RiboErase Globin (Roche, Basel, Switzerland). The quality controls were performed by fluorescence-based quantification method and fragment length analysis. The libraries were sequenced on a NovaSeq 6000 using 2x100 bp paired end reads. Bioinformatic analysis was performed with our in-house BITAP pipeline. In summary, the pipeline includes read filtering, alignment, somatic mutation calling, gene expression profiling, HLA typing, and peptide prediction and design. We follow the bioinformatics best practices neoantigen workflow combined with in-house ML-based model. WES quality control (QC) and preprocessing were perfomed with fastQC and skewer. Alignment was done by BWA-men against Hg38 GATK human genome, duplicates filtered with picard and Base Quality Recalibration score with GATK best practice. Variant calling was done with Mutect2 and annotated with VEP. Peptide binding affinity was calculated by mhcFlurry and immunogenicity ranking with BITAP AI model. HLA typing was obtained from tumor aligned data analysis with arcasHLA. Gene expression was obtained with RNAseq after alignment with STAR and gene expression calculation with RSEM. The peptide prediction was optimized by BITAP model.

**Peptide design and manufacturing**

The first peptide pool (BITAP-1) was generated by the evaluation of biopsies from bone metastases (**Supplementary Table 1**) which included different epitopes of both TSAs (tumor-specific antigens) and TAAs (tumor-associated antigens). The multi-epitope (class I and class II) peptides were connected using a cleavable linker (3-AA size). The peptides were then produced through chemical synthesis at GMP-like standard (>95% purity) to generate BITAP peptide pool together with XS15 as an adjuvant (14).

The second peptide pool (BITAP-2) was generated by the evaluation of biopsies from primary tumor and non-tumor tissues of the prostate (**Supplementary Table 2**), where non-tumor tissue was used as control to predict the TAAs. This pool included 7 peptides carrying 12 different epitopes of various TSAs (single nucleotide variant (SNV) and fusion), TAAs and cancer testis antigens.

**Analysis of T-cell Responses Using Ex Vivo stimulation and ELISpot Assay**

The potential of the peptides to induce a specific T-cell response in the T-cell repertoire of the patient was tested by ex vivo T-cell stimulation and subsequent ELISpot assay. This assay is used to determine the frequency of specific T cells by detecting specific cytokines, such as interferon-gamma (IFN-γ), secreted by individual immune cells in response to a particular peptide antigen. Since the frequencies of specific T cells are low prior to the immunization, PBMCs were pre-stimulated with a pool of all peptides for 12 to 13 days. The pre-stimulated PBMCs were plated into ELISpot plates coated with IFN-γ specific capture antibodies. The cells were incubated over night with the individual peptides, allowing antigen-specific immune responses to be captured and detected by the ELISpot assay (Mabtech, Nacka Strand, Sweden; used according to the manufacturers description). Pre-stimulated PBMC incubated without peptide were used as negative control. Responses were considered positive when the numbers of IFN-γ–secreting cells were at least 2-fold above the negative control. PBMCs were isolated prior to the BITAP administration. ELISpot assays were conducted using duplicate wells per peptide condition, and mean spot were reported.

# Supplementary Figures and Tables

## Supplementary Figures


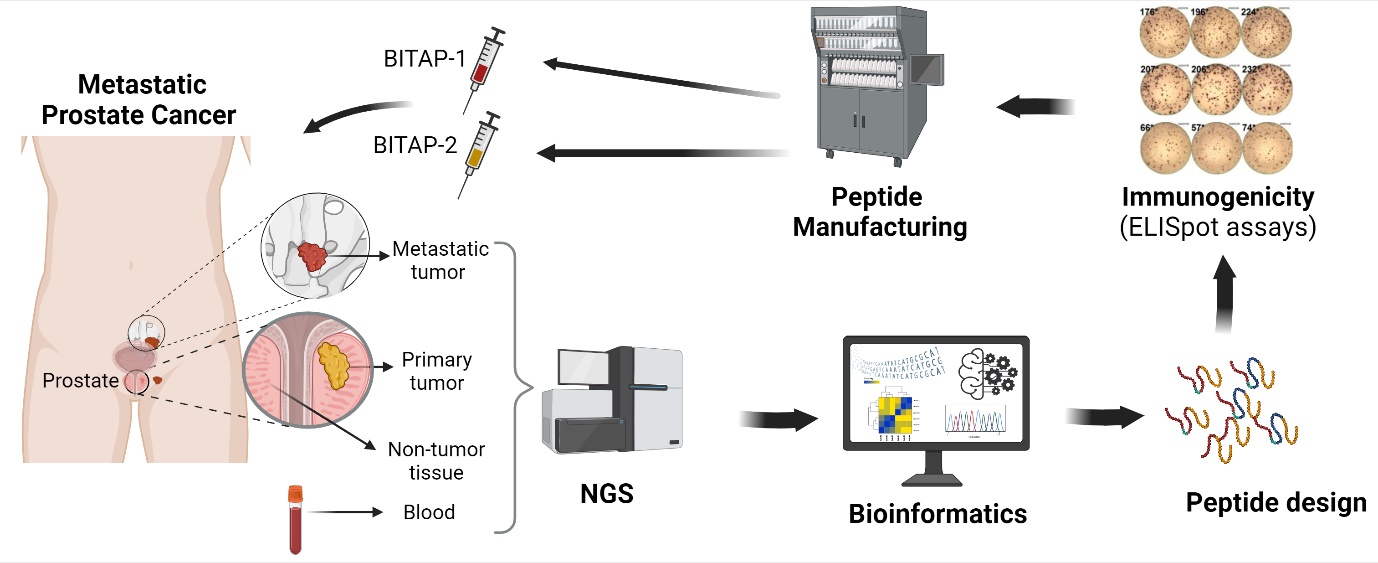


**Supplementary Figure 1**: Schematic overview of the workflow for BITAP generation. Created with BioRender.com.

## Supplementary Tables

**Supplementary Table 1**: List of manufactured peptides (BITAP-1)

| **ID** | **Peptide** | **gene** | **Antigen type** | **AA change** |
| --- | --- | --- | --- | --- |
| Peptide-01 | ETNFKSLFHDLFQKVEERQIKIWFQNRRMKWKK | SMC4 | TSA: SNV | V/F |
| Peptide-02 | IKPRILGFDTPHYWLCRQIKIWFQNRRMKWKK | KDM3A | TSA: SNV | S/R |
| Peptide-03 | NVGLSAVCTYNLSTAAAYCIHSINLHNFSNSVL | SEMA4D | TSA: SNV | A/T; R/C |
| Peptide-04 | MLFSNITPKAAYSPLTGGNMAFRQIKIWFQNRRMKWKK | TNC; LAMC1 | TSA: SNV | T/M; V/M |
| Peptide-05 | LSKNLVAQISALALQLAAYGNSITGIISSVLGHIS | RIF1 | TSA: SNV | L/V; G/S |
| Peptide-06 | KKIRKPRTIYSSLQLQAAAYGIYDALFDIESKVDPSK | DLX1; FOLH1 | TAA | --- |
| Peptide-07 | ESGCEERGAAMIQTV DPAAHRQIKIWFQNRRMKWKK | TMPRSS2-ERG | TSA: GF | --- |
| Peptide-08 | QRQLNTALPQPFREAPAYSNRQIKIWFQNRRMKWKK | PPFIA1-SHANK2 | TSA: GF | --- |
| Peptide-09 | KDYSVLYVVPGPVRFQRQIKIWFQNRRMKWKK | TMEFF2 | TAA | --- |
| Peptide-10 | LPVMLLIVARPVKLAAFPTSLSD | TMEFF2 | TAA | --- |
| Peptide-11 | LFKDLGLPARTVSTTFRQIKIWFQNRRMKWKK | DIP2A | TSA: SNV | A/T |
| Peptide-12 | RERRVASWAMSFERLRQIKIWFQNRRMKWKK | RGS12 | TSA: SNV | V/M |

**Table 2**: list of manufactured peptides (BITAP-2)

| **ID** | **Peptide** | **gene** | **Antigen type** | **AA change** |
| --- | --- | --- | --- | --- |
| Peptide-01 | RQRLALEAEKAAYEERGAAEALSVVRQIKIWFQNRRMKWKK | EXT2-CACNA2D1; TMPRSS2-ERG | TSA: GF | --- |
| Peptide-02 | IPDEYGNTALHYAIYNEDKLMRQIKIWFQNRRMKWKK | POTEG; POTEH | TAA | --- |
| Peptide-05 | LTPKKLQCVDLHVISNDRQIKIWFQNRRMKWKK | KLK3 | TAA | --- |
| Peptide-08 | SPRQCSSWTLAAYKLAAFPTSLRQIKIWFQNRRMKWKK | TMEFF2 | TAA | --- |
| Peptide-09 | HRCATITHLLAAYYLVLNFNKKRQIKIWFQNRRMKWKK | PTEN; ABLIM1 | TSA: FS, SNV | R/C |
| Peptide-10 | PPSNATAETQPIPQKAAYLEAIFLRDWDSLYSHDLDTSADS | LUZP2; PLD3 | TSA: SNV | E/Q; P/L |
| Peptide-12 | QSYPGSASLAAYSGMPRISKLRQIKIWFQNRRMKWKKKDEL | DXL1-FOLH1 | TAA | --- |
